# Risky Early Family Environment and Genetic Associations with Adult Metabolic Dysregulation

**DOI:** 10.3390/ijerph192114032

**Published:** 2022-10-28

**Authors:** Yazmine P. Huizar, Jenny M. Cundiff, Adam T. Schmidt, Matthew R. Cribbet

**Affiliations:** 1 Department of Psychology, Texas Tech University, Lubbock, TX 79409, USA; 2 Department of Psychology, University of Alabama, 348 Gordon Palmer Hall, Box 870348, Tuscaloosa, AL 35487, USA

**Keywords:** early risky family environment, genetic, body mass index, waist circumference

## Abstract

Growing up in a family environment characterized by neglectful parenting, overt conflict, and unsupportive relationships is associated with poor health in adulthood. A risky early family environment may also be associated with obesity in adulthood, likely through the activation of the HPA axis. Likewise, the GABAergic (gamma-aminobutyric acid) T>C single nucleotide polymorphism in the 1519 nucleotide position of the *GABAAα6* receptor subunit gene has been associated with a predisposition to a higher body mass index and a larger waist circumference. Participants (n = 213, M_age_ = 30.13 years, SD = 10.85; 57.7% men) from the Pittsburgh Cold Study 3 completed a demographic questionnaire, the Risky Families Questionnaire (RFQ) and had their height, weight, and waist circumference measured during a physical exam. Participant DNA was recovered from buccal swabs and genotyped for the various allelic types of the SNP according to published protocols. In secondary data analyses, we tested the hypothesis that early family environment and *GABRA6* would be positively associated with body mass index and waist circumference. We also examined diurnal cortisol as a mechanism linking both early risky family environment and *GABRA6* to metabolic outcomes. The findings provide evidence that a risky early family environment may exert more influence than genetic predisposition when determining the indices of metabolic health in adulthood.

## 1. Introduction

Obesity is on the rise in the United States, with around 42% of the adult population being classified as obese [1]. Obesity is a risk factor for several types of cancer, type II diabetes, heart disease, and all-cause mortality [2,3,4]. Additionally, these adverse health outcomes have a staggeringly negative economic effect, costing the U.S. alone billions of dollars a year on obesity-related medical care [5]. The role of genetics and stressful psychosocial experiences in childhood are factors associated with the development of obesity later in life. Identifying risk factors and how they are related to obesity is an important step toward preventing obesity and the associated negative physical health and financial outcomes.

Specific types of severe adverse childhood experiences have been linked to obesity and obesity-related diseases in adulthood [6]. For example, both physical abuse and experiencing domestic violence have shown a positive association with larger waist circumference (WC) and higher body mass index (BMI) [7,8]. Additionally, a meta-analysis revealed that childhood maltreatment was associated with an elevated risk of obesity over the course of life, and that this association remained after accounting for both adult and childhood socioeconomic status, alcohol intake, and physical activity [6]. However, less is known about whether milder, less severe forms of stress during childhood, such as growing up in an unsupportive, emotionally cold, and harsh family environment, also confer the risk for obesity in adulthood.

There is some evidence to suggest that growing up in an unsupportive, emotionally cold, and harsh family may be an important contributing factor to a higher BMI and a larger waist circumference, both of which are metabolic risk outcomes [9,10,11,12]. For example, growing up in an unsupportive, emotionally cold, and harsh, early family environment was directly associated with metabolic functioning in adulthood in a large epidemiological study [13]. A 1992 study supports this assertion, noting that a high-conflict family environment was associated with high cholesterol in males. Even when controlling for childhood BMI and sex, children who grew up with non-supportive parents were found to have an elevated risk of obesity in early adulthood [13].

According to theoretical models linking childhood stress and family dysfunction to adult health, children frequently exposed to a family environment characterized by conflict, aggression, and cold and unsupportive relationships may lead to adverse alterations in biological systems culminating in increased rates of a variety of health problems in adulthood [14]. In particular, children who grow up in “risky families” may experience an overactivation of the hypothalamic–pituitary–adrenal (HPA) axis [14]. During times of acute stress, HPA axis activation serves a protective function and promotes increased cardiovascular tone, respiratory rate, and metabolism, while constraining non-essential functions such as digestion, growth, and immunity [15]. However, prolonged activation of the HPA axis dysregulates these processes and contributes to negative health outcomes, such as insulin resistance and an increased propensity for abdominal obesity [16]. Thus, emerging evidence suggests that repeated exposure to stress during childhood may lead to prolonged activation of the HPA axis and, in turn, contribute to poor physical health in adulthood [17]. Direct tests of this hypothesis suggest that activation of the HPA axis may serve as a pathway linking more severe forms of childhood maltreatment, such as abuse and neglect, to BMI in adulthood, and this may generalize to less severe forms of family dysfunction as well [9,18].

However, environmental factors are not the sole contributors to obesity and obesity-related diseases, as genetic influences also play a significant role in their development. Obesity is now widely characterized as a complex disease controlled mostly by minor contributions from several genes interacting in tandem [19]. Therefore, a promising direction in the field of metabolic health research involves examining the contributing effects that single nucleotide polymorphisms (SNPs) have on the pathogenesis of obesity. SNPs are the most common genetic differences in humans, by some estimates accounting for 90% of all genetic variability [20]. SNPs are often studied through the candidate gene approach (CGA), which allows for a selective exploration of a gene or genomic regions of interest for a trait or disease risk based on a priori hypotheses. An integral advantage of this technique, when compared with studies with untargeted screenings (such as genome-wide association studies or GWAS), is their relative inexpensiveness and rapidity, and their emphasis on genes that have previously related to the disease. CGA studies are particularly useful in situations where allele frequencies are low, effect sizes are small, or the study population of interest is limited or unique [21]. CGA studies are also valuable for validating previous reports of genetic associations with the disease in different populations [21,22]. Nonetheless, this approach does have some limitations, including obstructing the discovery of new biological pathways, the reliance on previous knowledge about a gene, and its limited ability to include all possible causative genes [23,24,25].

The *GABRA6* gene is a member of the GABAergic receptor family that responds to GABA, the main inhibitory neurotransmitter of the central nervous system. A T>C polymorphism at nucleotide 1519 in the non-coding region of the *GABAAα6* receptor subunit gene means that there are several allelic variants of the *GABRA6* gene. *GABRA6* has been denoted as a polymorphism associated with hypercortisolism and abdominal fat deposition, establishing risk factors for morbidity and mortality attributable to obesity [26,27]. Carriers of the homozygous T/T or heterozygous T/C genotypes have been shown to exhibit a high waist-to-hip ratio (WHR), high abdominal sagittal diameter, as well as elevated diurnal cortisol secretion when compared to homozygous C/C carriers [28]. Excess concentrations of cortisol can also lead to elevated blood glucose levels, which over prolonged periods can cause insulin resistance, both of which have been linked to obesity [29].

The current study is a secondary data analysis of the Pittsburgh Cold Study 3, a quarantine study that examined factors for common cold susceptibility. One novel feature of the Pittsburgh Cold Study 3 was that it obtained retrospective measures of early family environmental stress along with genes that regulate target cell responsiveness to cortisol. In the present study, we examined the independent effects of growing up in a risky early family environment and *GABRA6* on BMI and WC in a community sample. We also examined diurnal cortisol secretion as a possible mechanism linking *GABRA6* and an early risky family environment to BMI and WC. Given that *GABRA6* (like all SNPs) remains stable across generations and that nearly half of the nation’s children have been found to have experienced at least one or more types of adversity, the current study provides novel insight into potential risk factors for metabolic dysregulation provided by less severe forms of childhood stressful experiences [30,31,32]. Thus, the purpose of this study is to test the hypothesis that an early risky family environment and *GABRA6* would be positively associated with BMI and WC and that these associations would be mediated through diurnal cortisol secretion.

## 2. Methods

### 2.1. Participants

Participants were recruited from the Pittsburgh, Pennsylvania greater metropolitan area through the use of newspaper advertisements. Informed consent was obtained from all participants prior to the start of the study (University of Pittsburgh IRB# 0701092). Six to eight weeks before the start of the study, participants were required to complete a telephone interview and undergo a physical examination to assess their health status. Participants were excluded if they had a previous nasal or otologic surgery; tested positive for the Human Immunodeficiency Virus (HIV); presented an abnormal profile of urinalysis, complete blood count, or blood enzyme levels; were pregnant or lactating; had a history of chronic illness (such as respiratory disorders, diabetes, or cardiovascular disease); or took certain types of medication regularly (such as antidepressants, sleeping pills, or tranquilizers). They were also excluded if they had been treated for a psychiatric illness the previous year or hospitalized for a psychiatric illness within the past five years. A total of 213 participants, 123 men and 90 women between the ages of 18 and 55 (M_age_ = 30.13 years, SD = 10.85; 57.7% men, 42.3% women), were evaluated and judged to be in good health. Upon the completion of the study, each participant was compensated for her or his participation. The data were collected by the Laboratory for the Study of Stress, Immunity, and Disease at Carnegie Mellon University under the directorship of Sheldon Cohen, PhD; and were accessed via the Common Cold Project website (www.commoncoldproject.com, accessed on 6 August 2020; grant number NCCIH ATOO6694).

### 2.2. Procedures

#### 2.2.1. Pre-Quarantine

Participants were interviewed in the evening over the phone consecutively over 14 days (10 weekdays and 4 weekend days) prior to viral exposure to obtain a daily assessment of their social interactions, mood, health behaviors, and physical symptoms. Multiple affective traits were assessed during the daily interviews, including depressive symptoms. The interviews lasted approximately 15 min, during which interviewers asked participants to rate, using a 5-point scale and a set of mood adjectives, how they had felt since awakening that day.

Genotyping. Approximately 7 to 8 weeks prior to quarantine, a sample collection was done via a buccal swab procedure on the inner check using a sterilized cytobrush (Histobrush, Hardwood Products Company, Guilford, ME, USA). Buccal cells were isolated by placing the swabs in a cryovial of 500 µL of 0.9% physiological saline and agitated. Subsequently, the swabs were pressed against a wall to release liquid and then removed from the tube [33]. This procedure was repeated three times in each cheek for each participant. The vials were stored at −80 °C until they were assayed for extraction of genomic DNA. A QIAamp DNA Mini Kit (QIAGEN, Valencia, CA, USA) was used for the extraction, a QIAGEN Repli-g Whole Genome Amplification Kit (GIAGEN) was used for amplification, and a Quant-iT™ PicoGreen^®^ dsDNA Assay Kit (Invitrogen, Carlsbad, CA, USA) was used for quantification [33]. Assaying was focused on the expression of the *GABRA6*, rs3219151 (1519 T>C, 3′-UTR) SNP.

Salivary Cortisol. Salivary cortisol samples were collected across multiple time points during the waking period of three non-consecutive days [34]. Six-to-one week prior to quarantine there was a 2-day repeated salivary cortisol collection in the participant’s natural environments (home, work, etc.). There was a 1-day repeated salivary cortisol collection during quarantine day 0 (baseline, prior to viral inoculation). For collection purposes, participants were provided with a plastic collection tube containing cotton rolls (Salivettes^®^; Sarstedt AG & Co., Nümbrecht, Germany). They were instructed to place the cotton in their mouth to saturate it with saliva then deposit the cotton back into the tube and reseal it. Participants were provided with written instructions and a handheld computer to signal collection times. The handheld computer provided a unique code for each collection, which participants were instructed to write on each sealed tube (along with the exact time and date of collection) and place it in their refrigerator. Participants were instructed to bring the tubes to their baseline study session to be collected by staff.

#### 2.2.2. During Quarantine

Participants were asked to complete self-report measures including the Risky Families Questionnaire (RFQ) during quarantine and after viral inoculation [35].

### 2.3. Measures

BMI and WC. Anthropometric data were assessed prior to the start of the two stress reactivity sessions. One session took place four to two weeks prior to quarantine, and the other session took place 4–6 weeks after the study. Measurements of height and weight were taken without the use of shoes or overgarments. Height was recorded to the nearest half-inch or half-centimeter, and weight was recorded to the nearest half-pound or half-kilogram. All English units were converted to metric. These data were used to calculate a participant’s body BMI using the following formula: weight (kg)/height (m)^2^. Waists were measured over a participant’s garments at the level of the navel. These data were used to calculate a participant’s waist/hip ratio with this formula: WC (cm)/hip circumference (cm). BMI and WC were averaged across the two reactivity sessions to increase reliability. Average values were used in all statistical analyses.

Risky Families Questionnaire (RFQ). Early family environment was measured by using a thirteen-item version of the RFQ to retrospectively capture the respondent’s family environment before they were 18 years of age [35]. The questionnaire employs a 5-point Likert scale, with each item ranging from 1 (not at all) to 5 (very often). Sample items include “Would you say the household you grew up in was chaotic and disorganized?” and “Would you say you were neglected while you were growing up, left on your own to fend for yourself?” Response options vary from 1 (indicating “rarely or none of the time”) to 5 (indicating “most or all of the time”). The RFQ has been validated against clinical interviews of childhood adverse experiences and demonstrated good internal consistency (Cronbach’s α ranges from 0.77–0.85) [35,36]. Previous research analyzing the PCS3 data have reported Cronbach’s α = 0.72 [37]. For the purposes of this study, higher total RFQ scores will be considered to reflect more stressful, less supportive early environments.

*GABRA6*. The *GABRA6* gene has an associated SNP involving a T to C substitution which results in several alleles: the homozygous C/C allele, the homozygous T/T allele, and the heterozygous T/C allele. Individuals carrying the T allele variants (either T/T or T/C alleles) are more likely to have higher BMI and WC along with excessive cortisol secretion.

Diurnal Cortisol. Salivary cortisol was measured seven times daily (1, 2, 4, 6, 8, 12, and 14 h after waking) during pre-quarantine days, and eight times during day 0 of quarantine (0, 1, 2, 4, 5, 7, 9, and 14 h after waking). Cortisol samples were assayed at the laboratory facilities of Dr. Clemens Kirschbaum in Dresden, Germany [34]. Cortisol concentrations were established through time-resolved fluorescence immunoassays using a cortisol-biotin conjugate as a tracer [38]. Only samples that were collected within ±45 min of the scheduled collection time were included for analysis; any samples collected outside of this time frame were treated as missing. The actual time rather than the expected time the participant provided for each cortisol sample was used to calculate both area under the curve (AUC) and slopes [39]. To calculate average diurnal cortisol levels, the AUC for each day was computed for individuals with sufficient data, which was defined as not having missed collection of any of the first three samples of the day (for steep diurnal rhythm) or missing more than two of the day’s remaining samples (for flat diurnal rhythm). The average total diurnal cortisol levels were calculated for participants who had data for a minimum of two of the three collection days by averaging total concentrations from all days with sufficient data [39]. Cortisol samples across the three study days were used to demonstrate participants’ typical diurnal cortisol secretion.

Covariates. Age, sex, race, and educational attainment (measured by total years of school completed) were obtained via self-report questionnaires and included as covariates.

Depressed Affect. The depressed affect was assessed using the depression subscale of the Negative Affect 2 component of the Daily Interview. Items for depressive symptoms include “sad” and “unhappy.”, The scale was created by calculating a mean score across the items for each of the 14 interview days. A 5-point scale was used to score the responses (0 = you have not felt that way at all today to 4 = you felt that way a lot today).

### 2.4. Overview of Analyses

Bivariate correlations were conducted to examine associations among study variables. Prior to conducting multiple linear regressions, preliminary analyses revealed no multicollinearity or issues with the independence of residuals but some departures from normality. Logarithmic transformations were applied to the outcome variables to correct violations of normality. Analyses were performed with and without transformed outcome variables. All models displayed a medium effect size prior to and subsequent to transformation (R^2^ = ~0.20). As the direction and significance of the associations did not change, analyses were conducted with untransformed variables to aid in the interpretation of the results.

A post hoc power analysis for multiple regression was conducted in G*power 3.1.9.7 to determine the detectable effect size of our results given our sample size. There was a total of seven predictors in the analyses for the first two hypotheses: early family environment and *GABRA6*, and the covariates: age, sex, race, and depression. Hypothesis three had a total of nine predictor variables: the first-order predictors and covariates, and the interaction between *GABRA6* and early family environment. In order to detect a small effect, the alpha was set to 0.05, power was set to 0.80, and Cohen’s f^2^ was set to 0.02 [40,41]. The power analysis indicated that a sample size of 725 participants would be required to detect a small effect. To determine a medium effect, the alpha was set to 0.05, the power was set to 0.80, and Cohen’s f^2^ was set to 0.15 [40,41]. The power analysis indicated that a sample size of 103 participants would be necessary to detect a medium effect. This study has a total of 213 participants, which allows us to detect a medium effect; however, the study is underpowered for detecting a small effect.

R version x64 3.4.3 was utilized to assess post hoc power for the mediation aim. The analysis was conducted using the powerMediation. VSMc function from the powerMediation package [42,43]. The sample size was designated as n and was set at 213 for all analyses. The b2 function indicates the regression coefficient for the mediator in the regression, the sigma.m function denotes the standard deviation of the mediator, the sigma.e function specifies the standard deviation of the random error term in the regression, and the corr.xm function represents the correlation between the specified predictor and the mediator. Alpha was set at 0.05 for all analyses. For the mediation with *GABRA6* as the predictor and BMI as the outcome, b2 was set at 0.0027, sigma.m was set at 215.8985, sigma.e was set at 5.9644, and corr.xm was set at −0.017. The resulting power for this mediation analysis was 0.2971. For the mediation with *GABRA6* as the predictor and WC as the outcome, b2 was set at 0.0082, sigma.m was set at 215.8985, sigma.e was set at 13.8464, and corr.xm was set at −0.017. The resulting power for this mediation analysis was 0.4625. For the mediation with the early family environment as the predictor and BMI as the outcome, b2 was set at 0.0030, sigma.m was set at 215.8985, sigma.e was set at 5.9086, and corr.xm was set at −0.072. The resulting power for this mediation analysis was 0.3581. For the mediation with *GABRA6* as the predictor and BMI as the outcome, b2 was set at 0.0087, sigma.m was set at 215.8985, sigma.e was set at 13.8576, and corr.xm was set at −0.072. The resulting power for this mediation analysis was 0.5053. None of the power analyses reached a power level of 0.8 or greater, indicating the mediation analyses were underpowered.

Multiple linear regressions were used to test associations among an early risky family environment and *GABRA6* with BMI and WC. The regression equations included *GABRA6* and early family environment as predictors, along with age, race, sex, and depressed affect as covariates. As *GABRA6* is a categorical variable, it was dummy coded to allow for inclusion in the regression analyses. The alleles for *GABRA6* were dummy coded as C/C = 0, T/C and C/C = 1. C/C was chosen as the reference category based on the higher incidence of hypercortisolism of the T/T and T/C carriers compared to C/C carriers [28].

For the mediation analyses, *GABRA6* was entered as a single multicategorical variable, as the PROCESS macro automatically creates dummy variables through its multicategorical option. Two separate mediation analyses were run: a heterozygous TC alleles comparison (comparing CC compared to TC) and a homozygous TT alleles comparison (CC compared to TT). The PROCESS macro, Model 4, for SPSS was used to investigate four separate hypotheses: (1) diurnal cortisol secretion will statistically mediate the association between early family environment and BMI; (2) diurnal cortisol secretion will statistically mediate the association between early family environment and WC; (3) diurnal cortisol secretion will statistically mediate the association between *GABRA6* and BMI; and (4) diurnal cortisol secretion will statistically mediate the association between *GABRA6* and WC [44].

The indirect effects were estimated through bias-corrected bootstrapped confidence intervals with 5000 iterations. This bootstrapping procedure uses the available sample and repeatedly resamples with replacements to create an empirical representation of the data [44]. This method estimates the indirect effects from the product of the a and b pathways 5000 times, orders them, and uses the lower 2.5% and upper 2.5% of these results as the boundaries of the 95% confidence interval. An indirect effect is achieved when the confidence intervals do not cross through zero.

## 3. Results

### 3.1. Correlation Analyses

Means, standard deviations, and correlations among study variables are presented in Table 1. As displayed in Table 1, age was significantly positively associated with both body mass index and waist circumference. Risky early family environment was significantly negatively associated with *GABRA6* and significantly positively associated with depressed affect.

### 3.2. Regression Analyses

As displayed in Table 2, controlling for age, sex, race, and depressed affect, a risky early family environment was significantly positively associated with BMI and WC, such that a more stressful, less supportive environment was associated with a higher BMI (*β* = 0.174, SE = 0.042, *t* = 2.637, *p* = 0.01) and a larger WC (*β* = 0.150, SE = 0.099, *t* = 2.335, *p* = 0.02). In regression analyses controlling for age, sex, race, and depressed affect, *GABRA6* was not significantly associated with either BMI (*β* = −0.105, SE = 0.587, *t* = −1.622 *p* = 0.11) or WC (*β* = −0.066, SE = 1.374, *t* = −1.051 *p* = 0.30). As displayed in Table 3, when a risky early family environment and *GABRA6*, along with age, sex, race, and depressed affect, were entered into the same model, a risky early family environment remained a significant predictor of BMI, (*β* = 0.166, SE = 0.042, *t* = 2.532, *p* = 0.01) WC (*β* = 0.1545, SE = 0.099, *t* = 2.260, *p* = 0.03), and *GABRA6* was not significantly associated with either BMI (*β* = −0.125, SE = 0.936, *t* = −1.968, *p* = 0.05) or WC (*β* = −0.076, SE = 2.217, *t* = −1.215, *p* = 0.23).

### 3.3. Mediation Analyses

**Figure 1:**
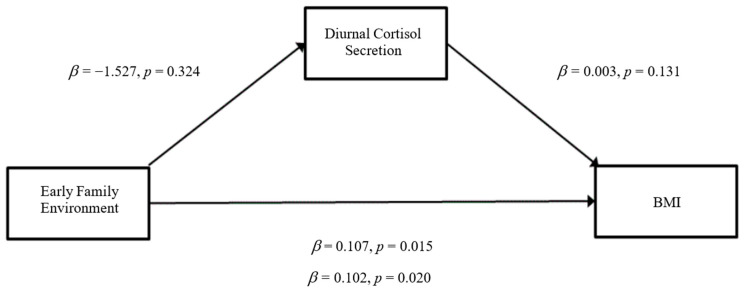
Mediation model for early risky family environment effect on BMI through the pathway of diurnal cortisol secretion (Model 4).

**Figure 2:**
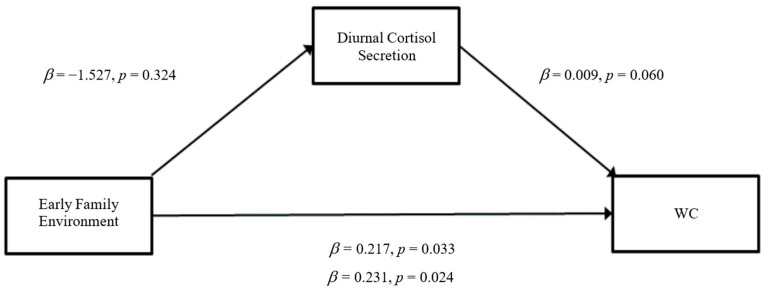
Mediation model for early risky family environment effect on WC through the pathway of diurnal cortisol secretion (Model 4).

**Figure 3:**
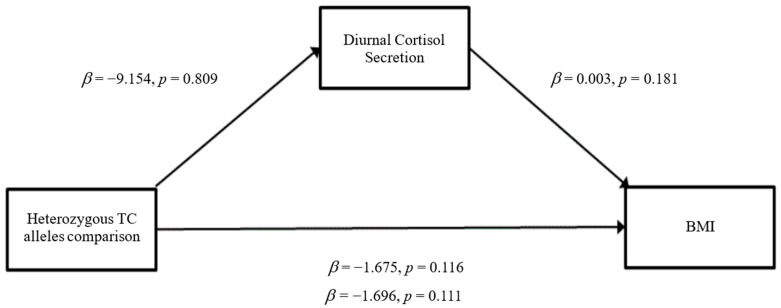
Mediation model for the Heterozygous TC alleles comparison with BMI through the pathway of diurnal cortisol secretion (Model 4).

**Figure 4:**
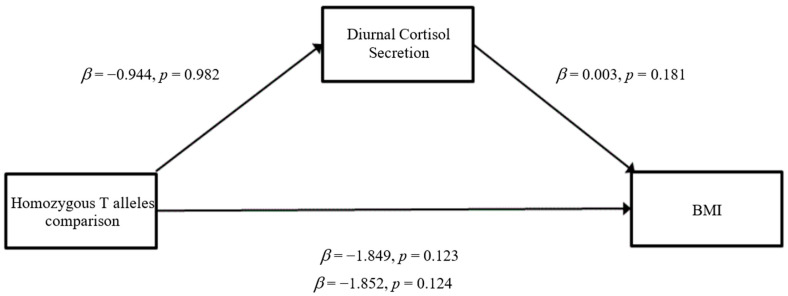
Mediation model for Homozygous T alleles comparison with BMI through the pathway of diurnal cortisol secretion (Model 4).

**Figure 5:**
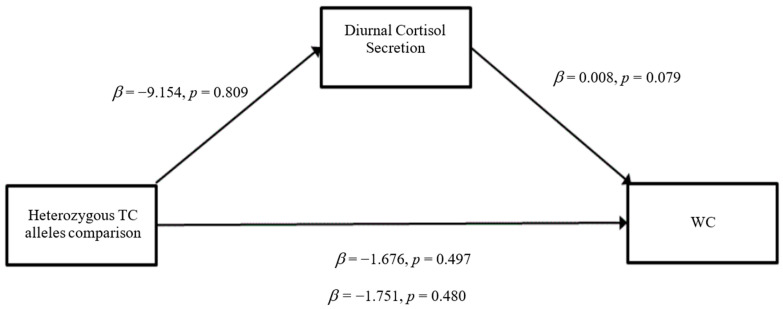
Mediation model for Heterozygous TC alleles comparison with WC through the pathway of diurnal cortisol secretion (Model 4).

**Figure 6:**
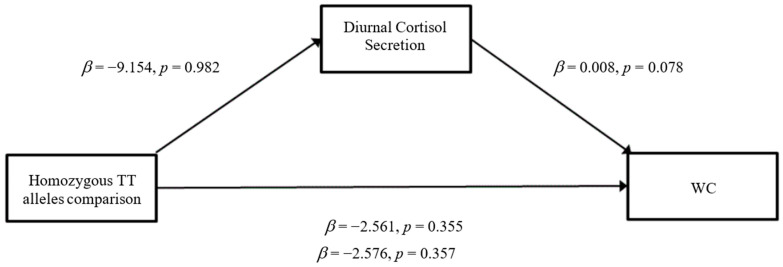
Mediation model for Homozygous TT alleles comparison with WC through the pathway of diurnal cortisol secretion (Model 4).

The results of the mediation analyses are presented in Figure 1, Figure 2, Figure 3, Figure 4, Figure 5 and Figure 6. For mediation analyses including *GABRA6* as a predictor variable, the reference category (the homozygous C/C allele) was compared against the homozygous T/T allele and the heterozygous T/C allele.

Risky Early Family Environment. When BMI was entered as the outcome variable, there were no significant direct effects of a risky early family environment on diurnal cortisol secretion (*β* = −1.526, SE = 1.542, *p* = 0.32) (Figure 1). There were no significant direct effects of diurnal cortisol secretion on BMI (*β* = 0.003, SE = 0.002, *p* = 0.18). However, significant direct effects were observed from a risky early family environment to BMI (*β* = 0.106, SE = 0.043, *p* = 0.02). Congruent with the regression analyses, a risky early family environment was statistically significant for the total effect path (*β* = 0.102, SE = 0.043, *p* = 0.02).

When WC was entered as the outcome variable, there were no significant direct effects of a risky early family environment on diurnal cortisol secretion (*β* = −1.527, SE = 1.543, *p* = 0.34) (Figure 2). Furthermore, there were no significant direct effects of diurnal cortisol secretion on WC (*β* = 0.008, SE = 0.005, *p* = 0.06). There were significant direct effects between a risky early family environment and WC (*β* = 0.231, SE = 0.101, *p* = 0.02). For the total effect model, a risky early family environment was statistically significant (*β* = 0.22, SE = 0.101, *p* = 0.03). Again, the mediation analyses did not indicate a significant indirect effect of a risky early family environment on WC through the pathway of diurnal cortisol secretion, indicating mediation had not occurred.

*GABRA6* When BMI was entered as the outcome variable, there were no significant direct effects of the heterozygous T/C alleles comparison on diurnal cortisol secretion (*β* = −9.154, SE = 37.901, *p* = 0.81) (Figure 3). The homozygous T/T alleles comparison was also not significantly associated with diurnal cortisol secretion (*β* = −0.944, SE = 42.716, *p* = 0.98) (Figure 4). Furthermore, there were no significant direct effects of diurnal cortisol secretion on BMI (*β* = 0.003, SE = 0.002, *p* = 0.18). The heterozygous T/C alleles comparison did not have any significant direct effects on BMI (*β* = −1.675, SE = 1.060, *p* = 0.12) nor did the homozygous T/T alleles comparison (*β* = −1.849, SE = 1.195, *p* = 0.123). For the total effect model, the heterozygous T/C alleles comparison did not show significant effects (*β*= −1.696, SE = 1.062, *p* = 0.11), nor did the homozygous T/T alleles comparison (*β* = −1.852, SE = 1.197, *p* = 0.12).

When WC was entered as the outcome variable, there were no significant direct effects of the heterozygous T/C alleles comparison on diurnal cortisol secretion (*β* = −9.154, SE = 37.901, *p* = 0.81) (Figure 5). Similar outcomes were observed for the homozygous T/T alleles comparison on diurnal cortisol secretion (*β* = −0.944, SE = 42.716, *p* = 0.98) (Figure 6). Furthermore, there were no significant direct effects of diurnal cortisol secretion on WC (*β* = 0.008, SE = 0.005, *p* = 0.08). The heterozygous T/C alleles comparison did not have any significant direct effects on WC (*β* = −1.676, SE = 2.461, *p* = 0.50) nor did the homozygous T/T alleles comparison (*β* = −2.568, SE = 2.774, *p* = 0.36). For the total effect model, the heterozygous T/C alleles comparison did not show significant effects (*β* = −1.751, SE = 2.475, *p* = 0.48), nor did the homozygous T/T alleles comparison (*β* = −2.576, SE = 2.789, *p* = 0.36). The mediation analyses did not indicate a significant indirect effect of *GABRA6* on BMI and WC through the pathway of diurnal cortisol secretion.

## 4. Discussion

The aims of the current study were to assess the contributions of *GABRA6* and a risky early family environment on BMI and WC in a community sample and to explore diurnal cortisol secretion as a pathway linking the association between *GABRA6* and a risky early family environment to BMI and WC.

Based on previous findings, it was hypothesized that a risky early family environment would be associated with BMI and WC [7,13,16]. After controlling for age, sex, race, and depression, the hypothesis was supported, as a risky early family environment was positively associated with both BMI and WC. Although prior work delineated the health consequences of severe family dysfunction, this study provided evidence that even milder forms of negative childhood exposures, including being raised in an unsupportive family environment that lacks parental warmth can be associated with adverse metabolic outcomes in adulthood.

Based on empirical evidence, it was hypothesized that those individuals with the T allele carriers of *GABRA6* would show positive and statistically significant associations with BMI and WC [26,28,45]. When controlling for age, sex, race, and depression, the hypothesis was not supported, as no significant association was found with any allele variant of *GABRA6*. The lack of support for the contribution of a single SNP for metabolic dysregulation, although surprising given previous research, is nonetheless congruent with the commonly accepted credence in genetic research that individual susceptibility to many diseases—including metabolic dysregulation—is a cumulative consequence derived from numerous low-penetrating genetic variables.

Although *GABRA6* has previously been linked with risk for metabolic dysregulation, making it an ideal gene to study under the CGA, the lack of significant associations with both BMI and WC could be explained by limitations inherent in the CGA, such as a relatively small sample size, low power, and low replicability [23,46]. As outlined by the power analyses, the study’s mediation analyses were underpowered and the regression analyses were unable to detect small effects, indicating that a larger sample size may be needed to detect significant effects. This is congruent with previous research, which has generally found that candidate–gene studies investigating different traits have been wanting. For example, an exhaustive review by Alghamdi and Padmanabhan (2014) found that only 6 out of 166 assumed associations were reliably replicated. The candidate gene approach has also been criticized for its inability to recognize additional functional variants due to complexity caused by phenotypic and locus heterogeneity and population stratification (differences in allele frequencies in a homogenous population) [47]. An unfortunate consequence of such a limitation is obtaining a potentially incomplete picture of disease pathology and precluding the discovery of new biological pathways [23,46].

Lastly, it was hypothesized that diurnal cortisol secretion would mediate the relationship between *GABRA6* alleles/early family environment and BMI/WC. When controlling for age, race, sex, and depression, the hypothesis was not supported. Mediation analyses indicated non-significant indirect effects of both *GABRA6* and a risky early family environment on BMI and WC through the pathway of diurnal cortisol secretion. Although previous work has found an association between diurnal cortisol secretion and markers of obesity the overall literature still shows mixed results between the link in diurnal cortisol profile and anthropomorphic measures of adiposity [18,45,48,49]. Clearly, more research is needed in this area to clarify this association.

### Limitations and Future Research

The results of this study must be viewed in the context of limitations that may inform future research. The sample size, although sufficiently large to detect psychosocial effects such as growing up in a risky early family environment, may be underpowered to detect genetic contributions from single SNPs, particularly those with low penetrance. Future studies seeking to assess genetic contributions from single SNPs may wish to employ a larger participant pool. As only one buccal swab was obtained from the participants, we are limited to analyzing only a single instance in time of the participant’s health, which may not be indicative of the participant’s fluctuating phenotypic profile with regard to potential epigenetic modifications impacting *GABRA6*. Due to retrospective reporting on the risky family questionnaire, there are inherent memory-related biases which call into question the reliability and validity of the participant’s long-term recall. In the future, researchers should consider replicating this study utilizing a longitudinal design in order to address this limitation. As the risky family questionnaire was completed after inoculation with the cold virus and during quarantine, the participant’s normative mood might have been altered, potentially impacting their memory recollection. Additionally, the generalizability of these findings is limited as the sample had relatively high education levels and lacked geographical diversity. Moving forward, it should be determined if these findings are similar in other samples, including clinical populations.

These limitations notwithstanding, the current study has several strengths. Importantly, this study adds to a growing literature demonstrating that growing up in a risky family can have negative implications for health. Specifically, this study examined body mass index and waist circumference, outcomes that are easy to obtain and associated with poor health such as cardiovascular disease, diabetes, and certain types of cancer. Despite not finding support for our mediator, cortisol was collected multiple times per day and averaged to increase the reliability of this measure. Moreover, we were able to demonstrate associations between a risky early family environment with WC and BMI in a healthy younger sample, removing any possible confounds that accompany older age and poor health.

## 5. Conclusions

In conclusion, we found that growing up in a risky early family environment was associated with higher BMI and WC during adulthood. These findings are important, as they clearly point to the importance of positive family relationships throughout childhood for health later in life [9]. Thus, it is likely that interventions, such as family therapy and parent–child interaction therapy, may not only be important for mental health, but for physical health outcomes as well. Future studies should examine how these interventions and others impact the physical health of at-risk children and their families.

The current findings suggest that developing an effective health improvement program requires a protracted, multilayered approach which includes interventions designed to help people change, implement, and maintain behaviors aimed at promoting a healthy family environment. Such interventions would ideally help parents identify the kinds of behaviors that have detrimental effects on a child’s behavioral and self-regulatory skills, or that are known to cause repeated incidences of stress.

## Figures and Tables

**Table 1:** Means, standard deviations, and zero-order correlations among study variables.

Variable	1	2	3	4	5	6	7
1. Age (years)		−0.114	−0.007	0.332 **	0.342 **	−0.108	0.055
2. Risky Early Family Environment			−0.168 *	0.108	0.083	0.306 **	−0.080
3. *GABRA6*				−0.118	−0.099	0.121	0.068
4. Body Mass Index (kg/m^2^)					0.894 **	−0.112	−0.082
5. Waist Circumference (cm)						−0.111	−0.017
6. Depressed Affect							−0.153 *
7. Diurnal Cortisol (nmol/L)							
Mean	30.13	28.23	7.28	27.46	88.93	0.957	3.70
Standard Deviation	10.85	10.24	1.82	6.49	15.72	1.07	0.181

Note: ** = *p* < 0.01; * = *p* < 0.05; Diurnal cortisol is expressed in log10 units for ease of interpretation.

**Table 2:** Summary of multiple regression analyses.

Outcome Variable BMI	*β (SE)*	*t*	*p*-Value
Model 1: Risky Early Family Environment	0.174 (0.042)	2.637	0.01
Age	0.307 (0.038)	4.814	0.00
Race	−0.160 (0.875)	−2.519	0.01
Sex	0.151 (0.823)	2.406	0.02
Depressed Affect	0.139 (0.399)	−2.118	0.04
Model 2: *GABRA6*	−0.066 (1.374)	−1.051	0.30
Age	0.413 (0.090)	6.606	0.00
Race	−0.144 (2.091)	−2.270	0.02
Sex	−0.098 (1.961)	−1.574	0.12
Depressed Affect	−0.041 (0.912)	−0.657	0.51
Outcome Variable WC			
Model 1: Risky Early Family Environment	0.150 (0.099)	2.335	0.02
Age	0.420 (0.090)	6.778	0.00
Race	−0.134 (2.063)	−2.163	0.03
Sex	−0.102 (1.939)	−1.672	0.10
Depressed Affect	−0.101 (0.941)	−1.570	0.12
Model 2: *GABRA6*	−0.105 (0.587)	−1.622	0.11
Age	0.295 (0.038)	4.582	0.00
Race	−0.165 (0.893)	−2.524	0.01
Sex	0.158 (0.837)	2.473	0.01
Depressed Affect	−0.072 (0.389)	−1.117	0.27

Note: BMI = Body Mass Index; WC = Waist Circumference; *GABRA6* = Gamma-Aminobutyric Acid Type A Receptor Alpha6 Subunit.

**Table 3:** Summary of multiple regression analysis with both a risky family environment and *GABRA6*.

Outcome Variable BMI	*β (SE)*	*t*	*p*
Risky Early Family Environment	0.166 (0.042)	2.532	0.01
*GABRA6*	−0.125 (0.936)	−1.968	0.05
Age	0.308 (0.038)	4.869	0.00
Race	0.142 (0.879)	2.223	0.03
Sex	0.164 (0.822)	2.615	0.00
Depressed Affect	−0.120 (0.401)	−1.807	0.07
Outcome Variable WC			
Risky Early Family Environment	0.145 (0.099)	2.260	0.03
*GABRA6*	−0.076 (2.217)	−1.215	0.23
Age	0.421 (0.090)	4.921	0.00
Race	0.123 (2.082)	1.966	0.05
Sex	−0.094 (1.94)	−1.539	0.13
Depressed Affect	−0.089 (0.951)	−1.368	0.17

Note: BMI = Body Mass Index; WC = Waist Circumference; *GABRA6* = Gamma-Aminobutyric Acid Type A Receptor Alpha6 Subunit.

## Data Availability

The data were collected by the Laboratory for the Study of Stress, Immunity, and Disease at Carnegie Mellon University under the directorship of Sheldon Cohen, PhD; and were accessed via the Common Cold Project website (www.commoncoldproject.com; grant number NCCIH AT006694).

## Abbreviations

WC = waist circumference; BMI = body mass index; *GABRA6* = Gamma-Aminobutyric Acid Type A Receptor Alpha6 Subunit.
